# Airway obstruction as the primary manifestation of infantile thyroid hemangioma

**DOI:** 10.1186/s13052-020-00904-6

**Published:** 2020-10-19

**Authors:** Yujian Liang, Ronghui Pu, Xueqiong Huang, Suping Li, Yingqian Chen, Wen Tang

**Affiliations:** 1 Department of Pediatric, The First Affiliated Hospital, Sun Yat-sen University, Guangzhou, P. R. China; 2 Department of Radiology, The First Affiliated Hospital, Sun Yat-sen University, Guangzhou, P. R. China

**Keywords:** Airway obstruction, Capillary, Infantile, Case report, Thyroid hemangioma

## Abstract

**Background:**

Thyroid hemangioma mostly occurs in adults and is extremely rare in infants. So far, only four pediatric cases of thyroid hemangioma have been reported, one of which has only been clinically diagnosed. Most of the reported cases are of cavernous hemangiomas; capillary hemangioma cases are very rare. To date, there are no reports on capillary thyroid hemangioma in an infant. Therefore, here we present the case of an infant with a primary capillary hemangioma of the thyroid gland, and discuss its treatment and follow-up.

**Case presentation:**

A2-month-old infant with normal thyroid function presented with airway obstruction as the primary manifestation of thyroid hemangioma. The main symptoms were laryngeal wheezing and dyspnea. Ultrasonography revealed hypoechoic lesions at the lower pole of the bilateral thyroid. Computed tomography revealed enlargement of the thyroid gland, inhomogeneous parenchyma enhancement, and multiple thyroid nodules. However, these imaging modalities were unable to provide an exact diagnosis and the nature of the mass remained unknown prior to an operation. Therefore, a postoperative histopathological examination was undertaken, which revealed capillary thyroid hemangioma. The symptoms significantly improved by a combined treatment involving surgery and oral propranolol.

**Conclusion:**

When a well-defined capsulate mass is detected on the medical image, the possibility of primary thyroid hemangioma must be considered.

## Background

Hemangiomas are the most common benign tumors in infants and young children, with an incidence of 4–5% [1]. While they can occur in any part of the body, they are more common in the skin and the subcutaneous tissues and are also observed in deep tissues such as the viscera, bones, muscles, etc. However, hemangiomas in the thyroid are rare. Because these tumors lack characteristic clinical manifestations and imaging signs, they are frequently misdiagnosed. In almost all patients, the diagnosis depends upon a postoperative histopathological examination [2]. Presently, cavernous hemangiomas are the most commonly reported, while capillary hemangioma cases are very rare. Here, we present the case of an infant with primary capillary hemangioma of the thyroid gland, and discuss its differential diagnosis, treatment, and follow-up.

This case report was approved by the Ethics Review Board of the First Affiliated Hospital, Sun Yat-sen University. Her parents provided written informed consent to publication of this report.

## Case presentation

**Fig. 1:**
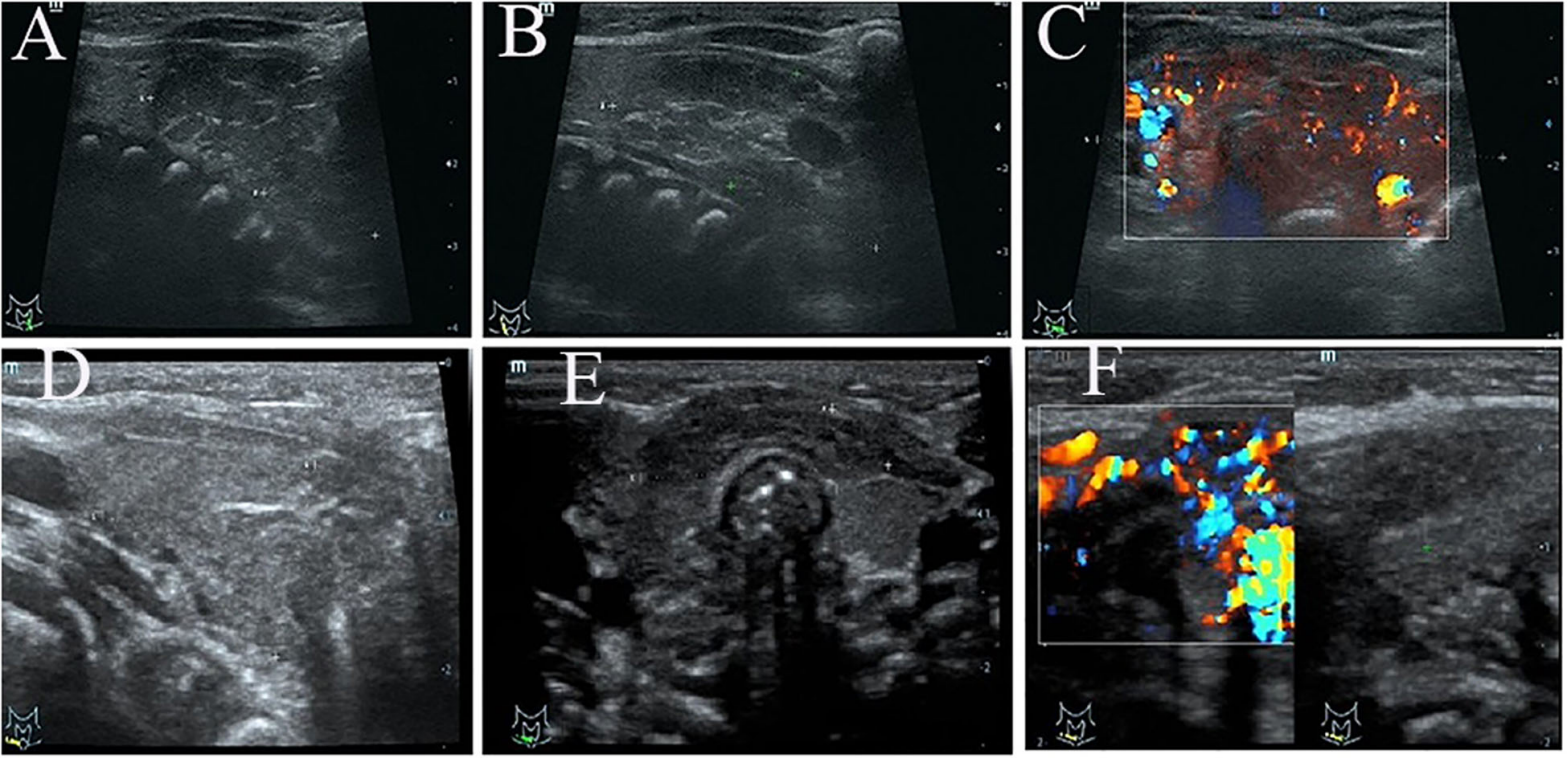
Ultrasound image of thyroid hemangioma. **a** Hypoechoic lesions are observed at the lower pole of the left thyroid and a chord-like hyperechoic lesion is observed within. **b** Hypoechoic lesions are observed at the lower pole of the right thyroid, and a chord-like hyperechoic lesion is observed within. **c** Hypoechoic lesions with a clear boundary and abundant blood supply are noted at the lower pole of the bilateral thyroid. **d**, **e**, **f** No recurrence of hemangioma was found

**Fig. 2:**
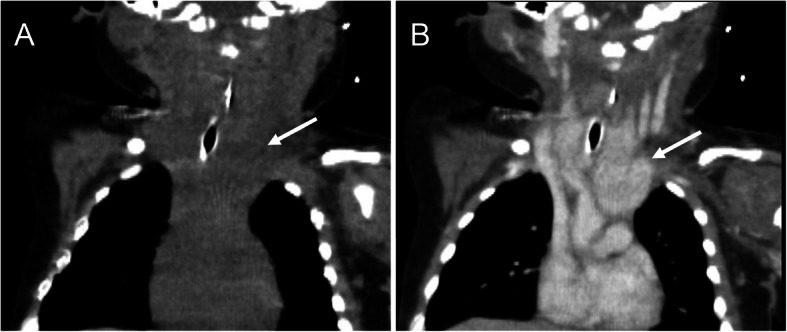
Non-enhanced and contrast-enhanced CT images of the thyroid hemangioma. **a** Non-enhanced CT imges show that the slightly hyperattenuated thyroid is enlarged and part of the left lobe stretches into the superior mediastinum. **b** In the contrast-enhanced CT image, the enlarged thyroid shows heterogeneous nodular enhancement

A 2-month-old baby girl was admitted to the hospital with recurrent laryngeal stridor and dyspnea lasting for more than 20 days. Before admission, she had undergone tracheal intubation for respiration support and received an anti-infective treatment from a local secondary level hospital. The patient’s dyspnea improved, but it was difficult to perform tracheal extubation. A computed tomography (CT) scan of the chest revealed pneumonia, consolidation in the right lung field, and a space occupying lesion in the thyroid. Due to difficulty of diagnosis and treatment at the local hospital, the patient was transferred to our first level hospital on Apr 28, 2019. The child was born at the second trimester with a gestational age of 41 + 3 weeks. Birth weight was 3.4 kg. The patient had no birth asphyxia, history of pathological jaundice, or any other family history of note. On physical examination after admission, heart rate was110–180 beats/min, blood pressure was67–104/38-61 mmHg, percutaneous oxygen saturation was95–98% (ventilator-supported breathing),no cervical or supraclavicular lymph nodes were palpated. The neck was symmetrical, without bulging in the anterior neck area; the overlying skin was not erythematous. The trachea was centered, the three depression signs of inspiration were observed, and coarse breath sounds without rales were noted on pulmonary auscultation. Laboratory investigations found that thyroid stimulating hormone (TSH) was 5.070uIU/mL (reference value 0.870–6.150uIU/mL), free triiodothyronine (FT3) was 4.700 pmol/L (reference value 3.800–6.000 pmol/L), free thyroxine (FT4)was13.900 pmol/L (referencevalue12.100–18.600 pmol/L),triiodothyronine(T3)was2.161 nmol/L (reference value1.34–2.70 nmol/L), thyroxine(T4)was97.4 nmol/L (reference value95 ~ 195 nmol/L), thyroglobulin antibody (TG-AB)was0.20Iu/ml (reference value was negative), thyroid peroxidase antibody(TPO-Ab)was12.1Iu/ml, alpha fetoprotein (AFP)was99.16μg/L (reference value was lessthan25ug/L), chorionic gonadotropinβ(B-HCG) was <1.20mIU/ml (reference value 0 ~ 3mIU/ml),ferritin (Fer) was 204.80μg/L (reference value194-238μg/L), specific neuronal enolase (NSE) was 150.50 ng/ml (reference value< 16.3 ng/ml). Arterial blood gas: PH 7.32,PCO2 43 mmHg,PO2 58 mmHg,P/F (PaO2/FiO2) 193 mmHg.An ultrasound scan of the thyroid (Fig. 1) revealed a hypoechoic lesion measuring 3.6 × 1.7 × 4.8 cm in diameter at the lower pole of the bilateral thyroid with an abundant blood supply. The boundary was clear, and a chord-like hyperechoic lesion was observed inside. The remaining thyroid gland showed a regular morphology, with a complete capsule and an even internal echo. The nature of the hypoechoic lesions remains to be determined. CT scanning of the thyroid with and without contrast (Fig. 2) revealed that the thyroid gland was enlarged and a part of the left lobe had stretched into the superior mediastinum. The thyroid parenchymal enhancement was inhomogeneous with multiple nodules. Chest CT revealed inflammation in the lower lobe of the left lung. No abnormality was found in abdominal ultrasound and cardiac ultrasound. The patient was given anti-infection treatment for pneumonia for 14 days after admission, pneumonia improved, however, an attempt to wean the patient off the invasive ventilator failed again (Arterial blood gas analysis under non-invasive ventilator assistance: PH 7.21,PCO2 74 mmHg,PO2 66 mmHg,P/F 165 mmHg). A multidisciplinary discussion led to a decision on surgical treatment. After completing the preoperative examination, the child underwent a total resection of the left thyroid gland and mass on May 8th.The size of the left thyroid and mass was about 3.0 × 3.0 × 1.0 cm.

**Fig. 3:**
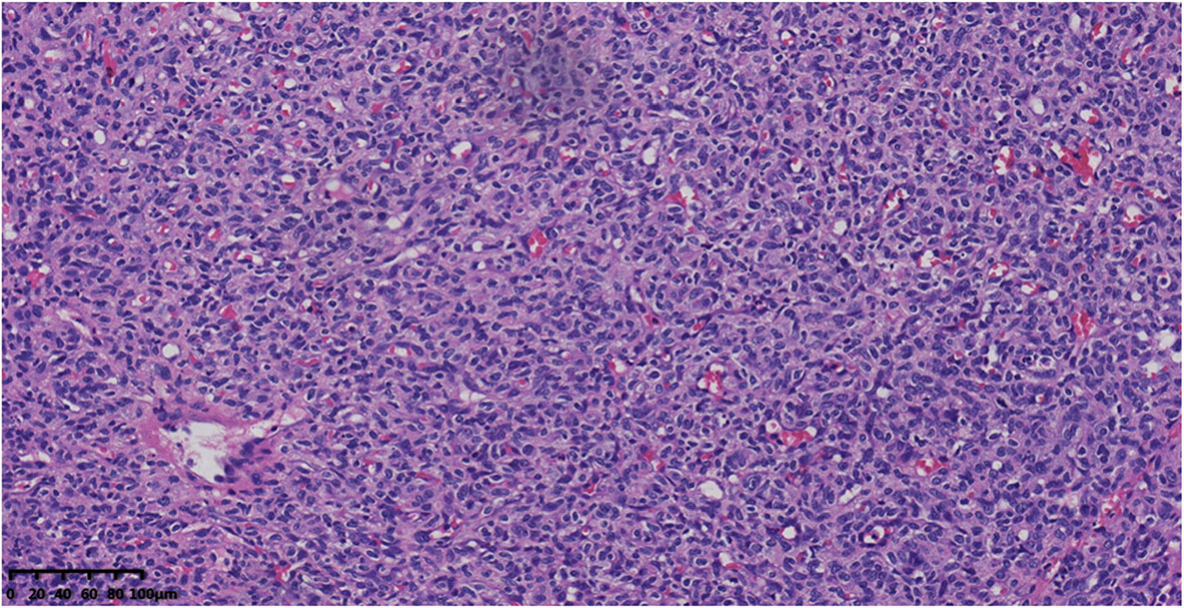
Specimen and histopathological picture of the excised thyroid mass. Capillary dilation can be seen, and solid cord changes are visible (H&E staining,40×)

Postoperative heart rate was 110–160 times/min, blood pressure was 67–89/38-53 mmHg, percutaneous oxygen saturation was 98–100%.Based on a postoperative pathological examination, infantile thyroid capillary hemangioma was diagnosed (Fig. 3). Then propranolol was administered orally (2.5 mg tid). There were no side effects of bradycardia, hypoglycemia, bronchospasm, or hyperkalemia during medication. A postoperative review showed that the thyroid function was normal, No recurrence of hemangioma was found by thyroid ultrasound (Fig. 1), the child’s respiration was smooth, and there was no laryngeal ringing after she was weaned from the ventilator 6 days after the operation. Arterial blood gas analysis after extubation: PH 7.38, PCO2 44 mmHg, PO2 176 mmHg,P/F 606 mmHg. The patient only had a small amount of hoarseness and low voice tone after stitch removal and extubation, possibly due to damage of the recurrent laryngeal nerve during the operation. The baby was discharged from hospital on May 20. During the subsequent six-month follow-up, the thyroid function remained normal and the tumor gradually shrank without recurrence; the hoarseness and low voice tone have also improved.

## Discussion and conclusion

A hemangioma originates from the mesodermal vascular endothelial cells. It undergoes rapid growth between one and 3 months after birth [3, 4], and can reach 80% of its final volume at the age of 3 months [5]. Hemangiomas rarely occur in the thyroid gland. Furthermore, they are mainly reported in adults and are rare in children. To the best of our knowledge, till 2019, only three cases have been reported in children ([6–8], Table 1). Frequent secondary venous stones in the lesion can lead to a stiffer texture, and imaging occasionally reveals clusters of strong echo or calcification. Hemangiomas are easily confused with malignant tumors. Even among experienced surgical experts, the preoperative diagnosis of thyroid hemangioma is quite difficult [2]. Fine needle puncture has a high risk of bleeding, and the final diagnosis in almost all patients depends upon a histopathological examination. Hemangioma is histologically classified as cavernous, capillary, synovial, venous, and so on. In case of thyroid hemangiomas, the cavernous type is the most commonly reported, while the capillary type is very rare. We present the first reported case of infantile capillary thyroid hemangioma with a review of literature.

**Table 1:** Case reports of thyroid hemangioma

Study, year	Age	Sex	Location	size (cm)	Symptoms	Diagnosis method/Histology	Treatment	Prognosis
Li Quanjiang, et al. [8], 2019	12Y	F	Right	2.0 × 1.0	none	Pathology	NM-	NM
Jacobson D, et al. [9], 2014	3 M	F	Right	3.1 × 3.7 × 2.2	dyspnea	Clinical diagnosis	propranolol	improve
Okuno, et al. [8], 1981	7Y	M	Diffuse	NM	none	Pathology/Capillary	NM -	NM
Ishida,et al. [7],1982	4Y	M	Isthmus	1.8 × 0.9 × 0.3	none	Pathology/Cavernous	NM -	NM
Our case	2 M	F	Both sides	3.0 × 3.0 × 1.0	dyspnea	Pathology/ Capillary	Operation and propranolol	improve

*Y* year, *M* month, *F* femal, *R* right, *NM* not mention

Often, thyroid hemangioma patients do not present with any specific clinical characteristics; sometimes, only a well-defined capsulate mass or growing mass is observed. In the present case, the main manifestation was airway obstruction without palpated mass and an absence of rythema on the neck skin. The mass was detected by CT and ultrasound examination. However, it is often difficult to distinguish such masses from malignant tumors using such preoperative imaging modalities. The diagnosis of capillary hemangioma was confirmed by surgical resection and pathological examination. A review of previous literature revealed only three pediatric cases of thyroid hemangioma. The children were aged between 3 months and 12 years [6–8]; one was an infant who had dyspnea and was placed on mechanical ventilation. Due to unstable vital signs, a pathological examination was not performed in this infant. Instead, the physicians relied on ultrasound, CT, and bronchoscopy for clinical diagnosis; the patient recovered after an empirical treatment with propranolol [8]. In case of the other two patients, no symptoms were reported and thyroid hemangioma was detected by routine medical examination (Table 1).

Because most infantile hemangiomas tend to regress spontaneously, active treatment is not recommended. Treatment is only required for complicated cases, which include life-threatening infantile hemangiomas (obstructive subglottic and bleeding gastrointestinal tumors, large hemangiomas causing cardiac insufficiency, and so on), infantile hemangiomas causing functional impairment (periocular hemangiomas causing amblyopia, obstructive tumors of the external auditory channel or the nose, and ulcerated infantile hemangioma), and infantile hemangiomas likely to cause disfigurement (large facial tumors, large infantile hemangiomas in the perimammary region in girls, and so on). Infantile hemangiomas that manifest as an obstruction require immediate therapy, such as in the present case. Treatment includes topical or intralesional injections, surgical resection, oral drugs, and new therapy methods. (Pharmacotherapy, for instance, especially laser therapy which is associated with a lower number of residual lesions as reported [9] and so on.)But oral propranolol at a dose of 2–3 mg/kg per day, according to guidelines,is the first choice, it should be administered as early as possible to avoid potential complications. Treatment is continued for at least 6 months and is often maintained until 12 months of age (occasionally longer) [3, 10]. Due to difficulties in preoperative diagnosis, most thyroid hemangiomas are only diagnosed by a postoperative histological examination. Therefore, surgical removal of thyroid hemangiomas is recommended. However, this carries a high risk, because hemangiomas are prone to hemorrhagingduring operation; around 2000 ml of blood loss is reported in adults [11]. Thus, it is important to maintain the integrity of the capsule, which can significantly reduce the risk of hemorrhaging and avoid a residual relapse. In our case, the nature of the mass was unclear before the operation, and its blood supply was rich. Fine needle aspiration could not provide enough tissue for pathological biopsy and carried a risk of bleeding. Therefore, this situation met the indication for operation and we performed a surgical treatment. The nature of the tumor was determined intraoperatively by a frozen pathological biopsy. Only the left lesion was removed, and the right side of the thyroid was preserved. Propranolol was administered after the surgery. Regular follow-up revealed that the hemangioma decreased in size. This indicated that surgical resection, combined with oral propranolol, may reduce hemorrhage risk and postoperative complications of thyroid hemangioma in infants; however, more studies are needed to confirm this.

To the best of our knowledge, this is the first reported case of a capillary thyroid hemangioma in an infant. In conclusion, when a well-defined capsulated mass is detected on the medical image, the possibility of primary thyroid hemangioma, in addition to that of a tumor, must be considered. The final diagnosis depends on a histopathological examination. When the tumor presses on the trachea and produces corresponding symptoms; it is an indication of surgical treatment. Although preoperative diagnosis is difficult, the prognosis after surgery is good. Surgical resection, combined with oral propranolol, reduces the surgical risk and postoperative complications of thyroid hemangioma in infants.

## Data Availability

The datasets used during the current study are available from the corresponding author on reasonable request.
